# Supplementary material for: Pyruvate Dehydrogenase Kinase 1 inhibition mediated oxidative phosphorylation enhancement in cartilage promotes osteoarthritis progression
Source: BMC Musculoskelet Disord. 2023 Jul 20;24:597. doi: 10.1186/s12891-023-06585-6 (PMC10357736; doi:10.1186/s12891-023-06585-6)
Supplement: Supplementary file 1 — Additional file 1. [file 12891_2023_6585_MOESM1_ESM.zip › KEGG.Gsea.1653622432464/KEGG_GAP_JUNCTION.html]

Details for gene set KEGG\_GAP\_JUNCTION[GSEA]

|  || Dataset | 55457\_collapsed\_to\_symbols.cls.cls #OA\_versus\_normal.cls.cls #OA\_versus\_normal\_repos |
| Phenotype | cls.cls#OA\_versus\_normal\_repos |
| Upregulated in class | normal |
| GeneSet | KEGG\_GAP\_JUNCTION |
| Enrichment Score (ES) | -0.20469049 |
| Normalized Enrichment Score (NES) | -0.8646849 |
| Nominal p-value | 0.7383628 |
| FDR q-value | 0.8714605 |
| FWER p-Value | 1.0 |
Table: GSEA Results Summary

  

Fig 1: Enrichment plot: KEGG\_GAP\_JUNCTION      
 Profile of the Running ES Score & Positions of GeneSet Members on the Rank Ordered List

  

| SYMBOL | TITLE | RANK IN GENE LIST | RANK METRIC SCORE | RUNNING ES | CORE ENRICHMENT || 1 | PDGFC | platelet derived growth factor C [Source:HGNC Symbol;Acc:HGNC:8801] | 103 | 0.911 | 0.0313 | No |
| 2 | DRD1 | dopamine receptor D1 [Source:HGNC Symbol;Acc:HGNC:3020] | 392 | 0.709 | 0.0394 | No |
| 3 | HTR2B | 5-hydroxytryptamine receptor 2B [Source:HGNC Symbol;Acc:HGNC:5294] | 562 | 0.636 | 0.0536 | No |
| 4 | MAPK3 | mitogen-activated protein kinase 3 [Source:HGNC Symbol;Acc:HGNC:6877] | 757 | 0.579 | 0.0635 | No |
| 5 | PRKACB | protein kinase cAMP-activated catalytic subunit beta [Source:HGNC Symbol;Acc:HGNC:9381] | 771 | 0.576 | 0.0873 | No |
| 6 | MAP2K5 | mitogen-activated protein kinase kinase 5 [Source:HGNC Symbol;Acc:HGNC:6845] | 1391 | 0.444 | 0.0581 | No |
| 7 | ADCY7 | adenylate cyclase 7 [Source:HGNC Symbol;Acc:HGNC:238] | 1408 | 0.442 | 0.0759 | No |
| 8 | ADCY1 | adenylate cyclase 1 [Source:HGNC Symbol;Acc:HGNC:232] | 1589 | 0.415 | 0.0798 | No |
| 9 | HTR2A | 5-hydroxytryptamine receptor 2A [Source:HGNC Symbol;Acc:HGNC:5293] | 1847 | 0.379 | 0.0761 | No |
| 10 | TUBA3E | tubulin alpha 3e [Source:HGNC Symbol;Acc:HGNC:20765] | 2169 | 0.342 | 0.0658 | No |
| 11 | PLCB4 | phospholipase C beta 4 [Source:HGNC Symbol;Acc:HGNC:9059] | 2217 | 0.337 | 0.0766 | No |
| 12 | EGF | epidermal growth factor [Source:HGNC Symbol;Acc:HGNC:3229] | 2399 | 0.315 | 0.0761 | No |
| 13 | TUBB7P | tubulin beta 7 pseudogene [Source:HGNC Symbol;Acc:HGNC:12413] | 2744 | 0.275 | 0.0611 | No |
| 14 | PDGFRA | platelet derived growth factor receptor alpha [Source:HGNC Symbol;Acc:HGNC:8803] | 2943 | 0.256 | 0.0567 | No |
| 15 | TUBB | tubulin beta class I [Source:HGNC Symbol;Acc:HGNC:20778] | 3296 | 0.221 | 0.0388 | No |
| 16 | GRM1 | glutamate metabotropic receptor 1 [Source:HGNC Symbol;Acc:HGNC:4593] | 3313 | 0.220 | 0.0470 | No |
| 17 | GNAI3 | G protein subunit alpha i3 [Source:HGNC Symbol;Acc:HGNC:4387] | 3694 | 0.186 | 0.0254 | No |
| 18 | PLCB1 | phospholipase C beta 1 [Source:HGNC Symbol;Acc:HGNC:15917] | 3724 | 0.183 | 0.0310 | No |
| 19 | PRKCA | protein kinase C alpha [Source:HGNC Symbol;Acc:HGNC:9393] | 3728 | 0.183 | 0.0387 | No |
| 20 | CDK1 | cyclin dependent kinase 1 [Source:HGNC Symbol;Acc:HGNC:1722] | 3744 | 0.182 | 0.0454 | No |
| 21 | PDGFD | platelet derived growth factor D [Source:HGNC Symbol;Acc:HGNC:30620] | 3850 | 0.171 | 0.0445 | No |
| 22 | HTR2C | 5-hydroxytryptamine receptor 2C [Source:HGNC Symbol;Acc:HGNC:5295] | 3873 | 0.167 | 0.0500 | No |
| 23 | GUCY1B1 | guanylate cyclase 1 soluble subunit beta 1 [Source:HGNC Symbol;Acc:HGNC:4687] | 3962 | 0.159 | 0.0500 | No |
| 24 | ITPR2 | "inositol 1,4,5-trisphosphate receptor type 2 [Source:HGNC Symbol;Acc:HGNC:6181]" | 4222 | 0.135 | 0.0356 | No |
| 25 | MAPK7 | mitogen-activated protein kinase 7 [Source:HGNC Symbol;Acc:HGNC:6880] | 4396 | 0.121 | 0.0274 | No |
| 26 | CSNK1D | casein kinase 1 delta [Source:HGNC Symbol;Acc:HGNC:2452] | 4529 | 0.111 | 0.0218 | No |
| 27 | TUBB2B | tubulin beta 2B class IIb [Source:HGNC Symbol;Acc:HGNC:30829] | 4777 | 0.089 | 0.0064 | No |
| 28 | GRM5 | glutamate metabotropic receptor 5 [Source:HGNC Symbol;Acc:HGNC:4597] | 4941 | 0.077 | -0.0030 | No |
| 29 | TUBAL3 | tubulin alpha like 3 [Source:HGNC Symbol;Acc:HGNC:23534] | 5043 | 0.070 | -0.0079 | No |
| 30 | ADCY9 | adenylate cyclase 9 [Source:HGNC Symbol;Acc:HGNC:240] | 5191 | 0.057 | -0.0169 | No |
| 31 | TUBA8 | tubulin alpha 8 [Source:HGNC Symbol;Acc:HGNC:12410] | 5208 | 0.056 | -0.0157 | No |
| 32 | TUBB1 | tubulin beta 1 class VI [Source:HGNC Symbol;Acc:HGNC:16257] | 5561 | 0.028 | -0.0420 | No |
| 33 | GRB2 | growth factor receptor bound protein 2 [Source:HGNC Symbol;Acc:HGNC:4566] | 5697 | 0.016 | -0.0519 | No |
| 34 | TUBB4A | tubulin beta 4A class IVa [Source:HGNC Symbol;Acc:HGNC:20774] | 5801 | 0.008 | -0.0595 | No |
| 35 | RAF1 | "Raf-1 proto-oncogene, serine/threonine kinase [Source:HGNC Symbol;Acc:HGNC:9829]" | 6187 | -0.019 | -0.0887 | No |
| 36 | GJD2 | gap junction protein delta 2 [Source:HGNC Symbol;Acc:HGNC:19154] | 6208 | -0.022 | -0.0894 | No |
| 37 | PRKCB | protein kinase C beta [Source:HGNC Symbol;Acc:HGNC:9395] | 6453 | -0.039 | -0.1067 | No |
| 38 | ADCY8 | adenylate cyclase 8 [Source:HGNC Symbol;Acc:HGNC:239] | 6646 | -0.053 | -0.1194 | No |
| 39 | GUCY1A2 | guanylate cyclase 1 soluble subunit alpha 2 [Source:HGNC Symbol;Acc:HGNC:4684] | 6731 | -0.058 | -0.1235 | No |
| 40 | ITPR1 | "inositol 1,4,5-trisphosphate receptor type 1 [Source:HGNC Symbol;Acc:HGNC:6180]" | 6752 | -0.059 | -0.1225 | No |
| 41 | MAPK1 | mitogen-activated protein kinase 1 [Source:HGNC Symbol;Acc:HGNC:6871] | 6853 | -0.068 | -0.1274 | No |
| 42 | PDGFRB | platelet derived growth factor receptor beta [Source:HGNC Symbol;Acc:HGNC:8804] | 6914 | -0.073 | -0.1289 | No |
| 43 | SRC | "SRC proto-oncogene, non-receptor tyrosine kinase [Source:HGNC Symbol;Acc:HGNC:11283]" | 6940 | -0.075 | -0.1276 | No |
| 44 | MAP3K2 | mitogen-activated protein kinase kinase kinase 2 [Source:HGNC Symbol;Acc:HGNC:6854] | 6976 | -0.078 | -0.1270 | No |
| 45 | PLCB2 | phospholipase C beta 2 [Source:HGNC Symbol;Acc:HGNC:9055] | 7052 | -0.084 | -0.1292 | No |
| 46 | PLCB3 | phospholipase C beta 3 [Source:HGNC Symbol;Acc:HGNC:9056] | 7118 | -0.090 | -0.1304 | No |
| 47 | NRAS | "NRAS proto-oncogene, GTPase [Source:HGNC Symbol;Acc:HGNC:7989]" | 7300 | -0.102 | -0.1401 | No |
| 48 | TUBB4B | tubulin beta 4B class IVb [Source:HGNC Symbol;Acc:HGNC:20771] | 7370 | -0.108 | -0.1409 | No |
| 49 | GNAQ | G protein subunit alpha q [Source:HGNC Symbol;Acc:HGNC:4390] | 7518 | -0.120 | -0.1472 | No |
| 50 | TUBA4A | tubulin alpha 4a [Source:HGNC Symbol;Acc:HGNC:12407] | 7668 | -0.132 | -0.1531 | No |
| 51 | LPAR1 | lysophosphatidic acid receptor 1 [Source:HGNC Symbol;Acc:HGNC:3166] | 7807 | -0.142 | -0.1577 | No |
| 52 | HRAS | "HRas proto-oncogene, GTPase [Source:HGNC Symbol;Acc:HGNC:5173]" | 7977 | -0.153 | -0.1643 | No |
| 53 | SOS2 | SOS Ras/Rho guanine nucleotide exchange factor 2 [Source:HGNC Symbol;Acc:HGNC:11188] | 8058 | -0.160 | -0.1637 | No |
| 54 | DRD2 | dopamine receptor D2 [Source:HGNC Symbol;Acc:HGNC:3023] | 8086 | -0.162 | -0.1588 | No |
| 55 | GNAI1 | G protein subunit alpha i1 [Source:HGNC Symbol;Acc:HGNC:4384] | 8110 | -0.164 | -0.1535 | No |
| 56 | ADRB1 | adrenoceptor beta 1 [Source:HGNC Symbol;Acc:HGNC:285] | 8212 | -0.172 | -0.1540 | No |
| 57 | TJP1 | tight junction protein 1 [Source:HGNC Symbol;Acc:HGNC:11827] | 8523 | -0.195 | -0.1697 | No |
| 58 | ADCY3 | adenylate cyclase 3 [Source:HGNC Symbol;Acc:HGNC:234] | 8572 | -0.199 | -0.1649 | No |
| 59 | PRKCG | protein kinase C gamma [Source:HGNC Symbol;Acc:HGNC:9402] | 8582 | -0.200 | -0.1570 | No |
| 60 | GNAI2 | G protein subunit alpha i2 [Source:HGNC Symbol;Acc:HGNC:4385] | 8686 | -0.208 | -0.1560 | No |
| 61 | GNAS | GNAS complex locus [Source:HGNC Symbol;Acc:HGNC:4392] | 8929 | -0.226 | -0.1651 | No |
| 62 | PRKG2 | protein kinase cGMP-dependent 2 [Source:HGNC Symbol;Acc:HGNC:9416] | 9126 | -0.242 | -0.1700 | No |
| 63 | GJA1 | gap junction protein alpha 1 [Source:HGNC Symbol;Acc:HGNC:4274] | 9325 | -0.258 | -0.1744 | No |
| 64 | GUCY1A1 | guanylate cyclase 1 soluble subunit alpha 1 [Source:HGNC Symbol;Acc:HGNC:4685] | 9420 | -0.266 | -0.1702 | No |
| 65 | ADCY2 | adenylate cyclase 2 [Source:HGNC Symbol;Acc:HGNC:233] | 9715 | -0.293 | -0.1805 | No |
| 66 | MAP2K2 | mitogen-activated protein kinase kinase 2 [Source:HGNC Symbol;Acc:HGNC:6842] | 9932 | -0.315 | -0.1838 | No |
| 67 | MAP2K1 | mitogen-activated protein kinase kinase 1 [Source:HGNC Symbol;Acc:HGNC:6840] | 10197 | -0.340 | -0.1897 | Yes |
| 68 | ITPR3 | "inositol 1,4,5-trisphosphate receptor type 3 [Source:HGNC Symbol;Acc:HGNC:6182]" | 10390 | -0.360 | -0.1892 | Yes |
| 69 | PRKACG | protein kinase cAMP-activated catalytic subunit gamma [Source:HGNC Symbol;Acc:HGNC:9382] | 10466 | -0.370 | -0.1791 | Yes |
| 70 | TUBB6 | tubulin beta 6 class V [Source:HGNC Symbol;Acc:HGNC:20776] | 10471 | -0.370 | -0.1634 | Yes |
| 71 | KRAS | "KRAS proto-oncogene, GTPase [Source:HGNC Symbol;Acc:HGNC:6407]" | 10544 | -0.377 | -0.1528 | Yes |
| 72 | PRKACA | protein kinase cAMP-activated catalytic subunit alpha [Source:HGNC Symbol;Acc:HGNC:9380] | 10611 | -0.384 | -0.1414 | Yes |
| 73 | PRKG1 | protein kinase cGMP-dependent 1 [Source:HGNC Symbol;Acc:HGNC:9414] | 10776 | -0.403 | -0.1368 | Yes |
| 74 | TUBA3C | tubulin alpha 3c [Source:HGNC Symbol;Acc:HGNC:12408] | 10884 | -0.415 | -0.1272 | Yes |
| 75 | TUBB2A | tubulin beta 2A class IIa [Source:HGNC Symbol;Acc:HGNC:12412] | 10972 | -0.427 | -0.1156 | Yes |
| 76 | PRKX | protein kinase X-linked [Source:HGNC Symbol;Acc:HGNC:9441] | 11634 | -0.522 | -0.1447 | Yes |
| 77 | TUBA1B | tubulin alpha 1b [Source:HGNC Symbol;Acc:HGNC:18809] | 11888 | -0.569 | -0.1399 | Yes |
| 78 | TUBA1A | tubulin alpha 1a [Source:HGNC Symbol;Acc:HGNC:20766] | 11974 | -0.589 | -0.1211 | Yes |
| 79 | ADCY6 | adenylate cyclase 6 [Source:HGNC Symbol;Acc:HGNC:237] | 12005 | -0.594 | -0.0979 | Yes |
| 80 | SOS1 | SOS Ras/Rac guanine nucleotide exchange factor 1 [Source:HGNC Symbol;Acc:HGNC:11187] | 12041 | -0.603 | -0.0746 | Yes |
| 81 | PDGFA | platelet derived growth factor subunit A [Source:HGNC Symbol;Acc:HGNC:8799] | 12104 | -0.621 | -0.0526 | Yes |
| 82 | EGFR | epidermal growth factor receptor [Source:HGNC Symbol;Acc:HGNC:3236] | 12109 | -0.622 | -0.0261 | Yes |
| 83 | TUBA1C | tubulin alpha 1c [Source:HGNC Symbol;Acc:HGNC:20768] | 12129 | -0.626 | -0.0006 | Yes |
| 84 | GNA11 | G protein subunit alpha 11 [Source:HGNC Symbol;Acc:HGNC:4379] | 12375 | -0.700 | 0.0105 | Yes |
| 85 | PDGFB | platelet derived growth factor subunit B [Source:HGNC Symbol;Acc:HGNC:8800] | 12389 | -0.704 | 0.0399 | Yes |
Table: GSEA details [plain text format]

  

Fig 2: KEGG\_GAP\_JUNCTION      
 Blue-Pink O' Gram in the Space of the Analyzed GeneSet

  

Fig 3: KEGG\_GAP\_JUNCTION: Random ES distribution      
 Gene set null distribution of ES for **KEGG\_GAP\_JUNCTION**
